# Daily use of high-potency cannabis is associated with more positive symptoms in first-episode psychosis patients: the EU-GEI case–control study

**DOI:** 10.1017/S0033291720000082

**Published:** 2021-06

**Authors:** Diego Quattrone, Laura Ferraro, Giada Tripoli, Caterina La Cascia, Harriet Quigley, Andrea Quattrone, Hannah E. Jongsma, Simona Del Peschio, Giusy Gatto, EU-GEI group, Charlotte Gayer-Anderson, Peter B. Jones, James B. Kirkbride, Daniele La Barbera, Ilaria Tarricone, Domenico Berardi, Sarah Tosato, Antonio Lasalvia, Andrei Szöke, Celso Arango, Miquel Bernardo, Julio Bobes, Cristina Marta Del Ben, Paulo Rossi Menezes, Pierre-Michel Llorca, Jose Luis Santos, Julio Sanjuán, Andrea Tortelli, Eva Velthorst, Lieuwe de Haan, Bart P. F. Rutten, Michael T. Lynskey, Tom P. Freeman, Pak C. Sham, Alastair G. Cardno, Evangelos Vassos, Jim van Os, Craig Morgan, Ulrich Reininghaus, Cathryn M. Lewis, Robin M. Murray, Marta Di Forti

**Affiliations:** 1 Social, Genetic and Developmental Psychiatry Centre, Institute of Psychiatry, Psychology and Neuroscience, King's College London, London, SE5 8AF, UK; 2 National Institute for Health Research (NIHR) Maudsley Biomedical Research Centre, South London and Maudsley NHS Foundation Trust, King's College London, London, UK; 3 Medical Faculty Mannheim, Central Institute of Mental Health, University of Heidelberg, Mannheim, Germany; 4 Department of Experimental Biomedicine and Clinical Neuroscience, University of Palermo, Via G. La Loggia 1, 90129 Palermo, Italy; 5 Department of Psychosis Studies, Institute of Psychiatry, Psychology and Neuroscience, King's College London, De Crespigny Park, Denmark Hill, London, SE5 8AF, UK; 6 Department of Chemical, Biological, Pharmaceutical and Environmental Sciences, University of Messina, 98100, Messina, Italy; 7 Psylife Group, Division of Psychiatry, University College London, 6th Floor, Maple House, 149 Tottenham Court Road, London, W1T 7NF, UK; 8 University of Bologna, 40126 Bologna, Italy; 9 Department of Health Service and Population Research, Institute of Psychiatry, Psychology and Neuroscience, King's College London, De Crespigny Park, Denmark Hill, London, SE5 8AF, UK; 10 Department of Psychiatry, University of Cambridge, Herchel Smith Building for Brain & Mind Sciences, Forvie Site, Robinson Way, Cambridge, CB2 0SZ, UK; 11 CAMEO Early Intervention Service, Cambridgeshire & Peterborough NHS Foundation Trust, Cambridge, CB21 5EF, UK; 12 Department of Medical and Surgical Science, Psychiatry Unit, Alma Mater Studiorum Università di Bologna, Viale Pepoli 5, 40126 Bologna, Italy; 13 Section of Psychiatry, Department of Neuroscience, Biomedicine and Movement, University of Verona, Piazzale L.A. Scuro 10, 37134 Verona, Italy; 14 INSERM, U955, Equipe 15, 51 Avenue de Maréchal de Lattre de Tassigny, 94010 Créteil, France; 15 Child and Adolescent Psychiatry Department, Institute of Psychiatry and Mental Health, Hospital General Universitario Gregorio Marañón, School of Medicine, Universidad Complutense, IiSGM, CIBERSAM, C/Doctor Esquerdo 46, 28007 Madrid, Spain; 16 Department of Medicine, Barcelona Clinic Schizophrenia Unit, Neuroscience Institute, Hospital Clinic of Barcelona, University of Barcelona, IDIBAPS, CIBERSAM, Barcelona, Spain; 17 Faculty of Medicine and Health Sciences – Psychiatry, Universidad de Oviedo, ISPA, INEUROPA. CIBERSAM, Oviedo, Spain; 18 Department of Preventative Medicine, Faculdade de Medicina FMUSP, University of São Paulo, São Paulo, Brazil; 19 EA 7280 Npsydo, Université Clermont Auvergne, Clermont-Ferrand, France; 20 Department of Psychiatry, Servicio de Psiquiatría Hospital ‘Virgen de la Luz’, Cuenca, Spain; 21 Department of Psychiatry, School of Medicine, Universidad de Valencia, Centro de Investigación Biomédica en Red de Salud Mental, Valencia, Spain; 22 Etablissement Public de Santé Maison Blanche, Paris, France; 23 Department of Psychiatry, Early Psychosis Section, Academic Medical Centre, University of Amsterdam, Amsterdam, The Netherlands; 24 Department of Psychiatry, Icahn School of Medicine at Mount Sinai, New York, New York, USA; 25 Department of Psychiatry and Neuropsychology, School for Mental Health and Neuroscience, South Limburg Mental Health Research and Teaching Network, Maastricht University Medical Centre, P.O. Box 616, 6200 MD Maastricht, The Netherlands; 26 National Addiction Centre, Institute of Psychiatry, Psychology & Neuroscience, King's College London, 4 Windsor Walk, London, SE5 8BB, UK; 27 Department of Psychology, University of Bath, 10 West, Bath, BA2 7AY, UK; 28 Department of Psychiatry, The University of Hong Kong, Hong Kong, China; 29 Li KaShing Faculty of Medicine, Centre for Genomic Sciences, The University of Hong Kong, Hong Kong, China; 30 Academic Unit of Psychiatry and Behavioural Sciences, University of Leeds, Leeds, UK; 31 Brain Centre Rudolf Magnus, Utrecht University Medical Centre, Utrecht, The Netherlands

**Keywords:** Cannabis use, cannabis-associated psychosis, psychopathology, psychotic experiences, symptom dimensions, first episode psychosis

## Abstract

**Background:**

Daily use of high-potency cannabis has been reported to carry a high risk for developing a psychotic disorder. However, the evidence is mixed on whether any pattern of cannabis use is associated with a particular symptomatology in first-episode psychosis (FEP) patients.

**Method:**

We analysed data from 901 FEP patients and 1235 controls recruited across six countries, as part of the European Network of National Schizophrenia Networks Studying Gene-Environment Interactions (EU-GEI) study. We used item response modelling to estimate two bifactor models, which included general and specific dimensions of psychotic symptoms in patients and psychotic experiences in controls. The associations between these dimensions and cannabis use were evaluated using linear mixed-effects models analyses.

**Results:**

In patients, there was a linear relationship between the positive symptom dimension and the extent of lifetime exposure to cannabis, with daily users of high-potency cannabis having the highest score (*B* = 0.35; 95% CI 0.14–0.56). Moreover, negative symptoms were more common among patients who never used cannabis compared with those with any pattern of use (*B* = −0.22; 95% CI −0.37 to −0.07). In controls, psychotic experiences were associated with current use of cannabis but not with the extent of lifetime use. Neither patients nor controls presented differences in depressive dimension related to cannabis use.

**Conclusions:**

Our findings provide the first large-scale evidence that FEP patients with a history of daily use of high-potency cannabis present with more positive and less negative symptoms, compared with those who never used cannabis or used low-potency types.

## Introduction

There is compelling evidence to suggest that cannabis use is associated with psychotic disorders (Marconi, Di Forti, Lewis, Murray, & Vassos, 2016). However, it is unclear whether cannabis use is a ‘modifier’ factor that affects symptom presentation of psychotic disorders. The existence of a pattern of psychotic symptomatology particularly associated with cannabis has been described in several case series (Bernhardson & Gunne, 1972; Chopra & Smith, 1974; Spencer, 1971; Talbott & Teague, 1969; Bromberg, 1934). Nevertheless, case and cohort studies have found mixed results as to whether (Addington & Addington, 2007; Bersani, Orlandi, Kotzalidis, & Pancheri, 2002; Foti, Kotov, Guey, & Bromet, 2010; Grech, Van Os, Jones, Lewis, & Murray, 2005; Green et al., 2004; Negrete, Knapp, Douglas, & Smith, 1986; Peralta & Cuesta, 1992; Ringen et al., 2016; Seddon et al., 2016) or not (Barrowclough, Gregg, Lobban, Bucci, & Emsley, 2015; Boydell et al., 2007; Dubertret, Bidard, Ades, & Gorwood, 2006; Stirling, Lewis, Hopkins, & White, 2005; Thornicroft, Meadows, & Politi, 1992; Tosato et al., 2013; van Dijk, Koeter, Hijman, Kahn, & van den Brink, 2012) psychotic patients using cannabis present with more positive symptoms than those not using cannabis. Moreover, there is mixed evidence of any relationship between cannabis use and negative symptoms in psychosis. Some reports suggest fewer negative symptoms in patients with psychosis who use cannabis (Bersani et al., 2002; Green et al., 2004; Peralta & Cuesta, 1992), which is consistent with having enough social skills to obtain the substance (Murray et al., 2017). However, this association has not been confirmed in other studies (Grech et al., 2005; Seddon et al., 2016) and others even reported a positive association (Ringen et al., 2016).

These inconsistencies might be explained by differences in study design and methods. For example, only a few findings were based on first-episode psychosis (FEP) patients (Addington & Addington, 2007; Grech et al., 2005; Seddon et al., 2016; Tosato et al., 2013), which minimize selection and recall bias, and the confounding effect of antipsychotic drugs on symptoms. In addition, a meta-analysis of longitudinal studies concluded that most examinations lacked sufficient power to detect an effect of cannabis on symptoms, or inadequately controlled for potential confounders (Zammit et al., 2008). Furthermore, although a few studies included information on the frequency of use, all failed to obtain detailed information on the lifetime pattern of cannabis use, especially on the type and strength of cannabis used. Of note, potent cannabis varieties, with high concentrations of Delta-9-Tetrahydrocannabinol (Δ9-THC), have been associated with the most harm to mental health (Di Forti et al., 2015; Freeman et al., 2018) and, in recent years, these potent types have become more available worldwide (ElSohly et al., 2016; Freeman et al., 2019; Potter, Hammond, Tuffnell, Walker, & Di Forti, 2018). Finally, no studies have used factor analysis of observed symptoms to evaluate to what extent cannabis use is a factor influencing the clinical heterogeneity of psychosis.

On the other hand, in the general population, there are consistent findings regarding the association between cannabis use and psychotic experiences (Ragazzi, Shuhama, Menezes, & Del-Ben, 2018). However, most studies had limited geographical coverage and the examined population was scarcely representative of the population at risk of psychosis (Ragazzi et al., 2018).

In this study, we set out to clarify the association between detailed patterns of cannabis use and transdiagnostic symptom dimensions in a large multinational FEP sample. In addition, we examine the association between detailed patterns of cannabis use and subclinical symptom dimensions in a large sample of controls representative of the population at risk in each catchment area.

Specifically, we sought to test the hypotheses that: (1) positive psychotic symptoms are more common among FEP patients with more frequent lifetime use of cannabis and greater exposure to high-potency varieties; (2) positive psychotic experiences are more common in population controls with a recent use of cannabis, who would be more resilient to the long-term effects of cannabis; (3) negative symptoms are more common among those patients who have never used cannabis.

## Methods

### Study design and participants

This analysis is based on the incidence and case–control study work package of the EUropean network of national schizophrenia networks studying Gene-Environment Interactions (EU-GEI).

FEP individuals were identified between 2010 and 2015 across six countries to examine the incidence rates of schizophrenia and other psychotic disorders (Jongsma et al., 2018), and symptomatology at psychosis onset (Quattrone et al., 2019). To examine the risk factors, we sought to perform an extensive assessment on approximately 1000 FEP patients and 1000 population-based controls during the same time period.

Patients were included in the case–control study if they met the following criteria during the recruitment period: (a) aged between 18 and 64 years; (b) presentation with a clinical diagnosis for an untreated FEP, even if longstanding [International Statistical Classification of Diseases and Related Health Problems, Tenth Revision (ICD-10) codes F20–F33]; (c) resident within the catchment area. Exclusion criteria were: (a) previous contact with psychiatric services for psychosis; (b) psychotic symptoms originating from an identified organic condition; and (c) transient psychotic symptoms resulting from acute intoxication (ICD-10: F1x.5).

The recruitment of controls followed a mixture of random and quota sampling methods, in order to achieve the best possible representativeness in age, sex and ethnicity of the population living in each catchment area. The identification process varied by site and was based on locally available sampling frames, including mostly the use of lists of all postal addresses and general practitioners' lists from randomly selected surgeries. When these resources were not fully available, Internet and newspapers advertising were used to fill quotas. Exclusion criteria for controls were: (a) diagnosis of a psychotic disorder; (b) ever having been treated for psychotic symptoms.

We analysed data from 11 catchment areas, including urban and less urban populations [i.e. Southeast London, Cambridgeshire and Peterborough (England); central Amsterdam, Gouda and Voorhout (the Netherlands); Bologna municipality, city of Palermo (Italy); Paris (Val-de-Marne), Puy-de-Dôme (France); Madrid (Vallecas), Barcelona (Spain); and Ribeirão Preto (Brazil)]. Further information on the case–control sample and the recruitment strategies is included in the online Supplementary material.

### Measures

Data on age, sex and ethnicity were collected using a modified version of the Medical Research Council Sociodemographic Schedule (Mallett, 1997). The OPerational CRITeria (OPCRIT) system (McGuffin, Farmer, & Harvey, 1991) was used by centrally trained investigators, whose reliability was assessed before and throughout the study (*k* = 0.7), to assess psychopathology in the first 4 weeks after the onset and generate research-based diagnoses based on different diagnostic classification systems. The Community Assessment of Psychic Experiences (CAPE) (Stefanis et al., 2002) was administered to controls to self-report their psychotic experiences. The reliability of the CAPE is good for all the languages spoken in the countries forming part of the EU-GEI study (http://cape42.homestead.com).

A modified version of the Cannabis Experience Questionnaire (CEQ_EU-GEI_) (Di Forti et al., 2009) was used by investigators to collect extensive information on the patterns of use of cannabis and other drugs. We used six measures of cannabis use (online Supplementary Table S2), including a variable measuring specific patterns of cannabis exposure by combining the frequency of use with the potency of cannabis. As illustrated in the Supplementary material, the cannabis potency variable was based on the data published in the European Monitoring Centre for Drugs and Drug Addiction (EMCDDA) (Di Forti et al., 2019; European Monitoring Centre for Drugs & Drug Addiction, 2013).

We selected confounders based on their possible association with cannabis use and/or symptom dimensions. These included: sex; age; ethnicity; use of stimulants, hallucinogens, ketamine, cocaine, crack and novel psychoactive substances; current use of tobacco cigarettes (smoking 10 cigarettes or more per day = 1); and current use of alcohol (drinking 10 alcohol units or more per week = 1).

### Statistical analysis

#### Dimensions of psychotic symptoms in patients and psychotic experiences in controls

Data from OPCRIT and CAPE were analysed using multidimensional item response modelling in M*plus*, version 7.4 (Muthén & Muthén, 2012), to estimate two bifactor models, based on the associations among observer ratings of psychotic symptoms in patients and self-ratings of psychotic experiences in controls. This methodology is described in full in our EU-GEI paper on symptom dimensions in FEP patients (Quattrone et al., 2019), and it was likewise applied to psychotic experiences in population controls. Briefly, CAPE items were dichotomized as 0 ‘absent’ or 1 ‘present’. In order to ensure sufficient covariance coverage for item response modelling, we used items with a valid frequency of ‘present’ ⩾10% in our sample, and we excluded items with low correlation values (<0.3) based on the examination of the item correlation matrix. As in the previous analysis in patients, the bifactor solution was compared with other solutions (i.e. unidimensional, multidimensional and hierarchical models) using Log-Likelihood (LL), Akaike Information Criterion (AIC), Bayesian Information Criterion (BIC) and Sample-size Adjusted BIC (SABIC) as model fit statistics. Path diagrams that illustrate these models are presented in online Supplementary Fig. S1. Reliability and strength indices such as McDonald's omega (*ω*) (Rodriguez, Reise, & Haviland, 2016), omega hierarchical (*ω*_H_) (Rodriguez et al., 2016) and index *H* (Hancock & Mueller, 2001) were computed to determine: (1) the proportion of common variance accounted by general and specific symptom dimensions; (2) the proportion of reliable variance accounted by the general dimension not unduly affected by the specific dimensions; (3) the proportion of reliable variance accounted for by each specific dimension not unduly affected by the general and all the other specific dimensions; (4) the overall reliability and replicability of the bifactor construct of psychosis-like experiences. Finally, we generated factor scores for one general psychotic experience dimension and three specific dimensions of positive, negative and depressive psychotic experiences.

For patients, we used the previously generated factor scores for one general psychosis dimension and five specific dimensions of positive, negative, disorganized, manic and depressive symptoms (Quattrone et al., 2019).

#### Symptom dimensions and cannabis use

We evaluated the relationship between psychotic symptom dimensions in patients, or psychotic experience dimensions in controls, and cannabis use using linear mixed-effects models in STATA14 (StataCorp, 2015). We specifically modelled symptom dimension scores as a function of each of the six measures of cannabis use. We then evaluated the combined effect of frequency of use and potency of cannabis. To account for the non-independence of symptom profiles of subjects assessed within the same country (e.g. due to cultural similarities), and for the potential within-site correlation (e.g. due to context factors), we fitted a three-level mixed model, where the random effect encompassed two levels of random intercepts: one due to the countries, and another due to the sites within the countries. Finally, we used the Benjamini–Hochberg (B-H) procedure to reduce the false discovery rate, which we set at 5%.

## Results

### Sample characteristics

We analysed data from 901 FEP patients and 1235 controls. The main socio-demographic characteristics and history of substance misuse of patients and controls are presented in online Supplementary Table S1. Online Supplementary Tables S3 and S5 show the sample prevalence of psychotic experiences in controls and of psychotic symptoms in patients.

### Bifactor model of psychotic experiences in controls

**Fig. 1.**
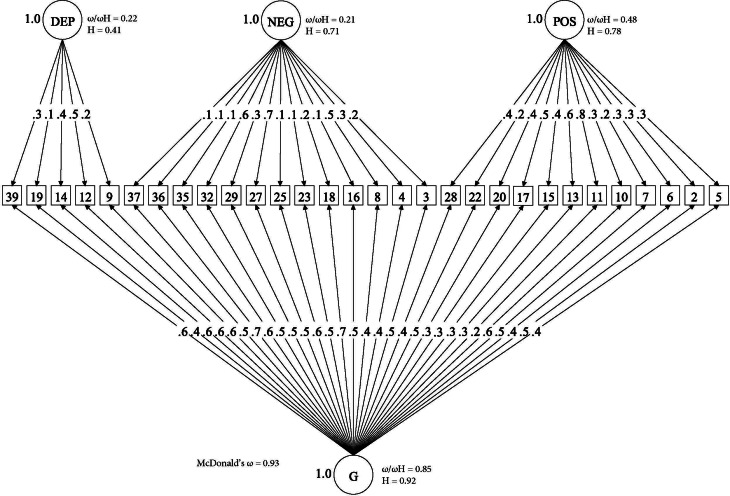
Bifactor model of psychotic experiences in controls. (□) Observed variables (No. of CAPE items); (○) Unobserved variables (latent factors); (→) standardized item loading estimation onto latent factors; G, general psychosis-like factor; Specific psychotic experiences factors: DEP, Depression; NEG, Negative; POS, Positive. Reliability and strength estimates: H = construct reliability index; *ω* = McDonald omega; *ω*_H_ = hierarchical omega; *ω*/*ω*_H_ = Relative omega. Explanatory note: McDonald‘s *ω* is an estimate of the proportion of the common variance accounted by general and specific symptom dimensions (Rodriguez et al., 2016). Relative omega (*ω*/*ω*_h_) is the amount of reliable variance explained in the observed scores attributable to (*a*) the general factor independently from the specific symptom dimensions, and (*b*) each specific symptom dimension independently from the general factor. *H* is an index of the quality of the measurement model based on the set of CAPE items for each dimension (Hancock & Mueller, 2001). Indices can range from 0 to 1, with values closer to 1 indicating a better construct reliability and replicability across studies.

Online Supplementary Table S4 shows that, as in our previous analysis of the OPCRIT items (Quattrone et al., 2019), the bifactor model provided the best fit for the CAPE items, as illustrated by AIC, BIC and SABIC substantially lower compared with competing models. This solution explained 60% of the unique variance. In addition, Fig. 1 shows that, within the bifactor model, the explained variance was due to individual differences mostly on the general psychotic experience dimension. This is illustrated by the relative *ω* coefficient, which, for example, showed that 85% of the reliable variance was due to the general dimension when partitioning out the variability in scores due to the specific dimensions. Moreover, factor loadings of moderate to high magnitude were observed for most items on the general psychotic experience dimension, whereas factor loadings of a smaller magnitude were observed for the specific dimensions (Fig. 1). Consistently, the index *H*, which is a measure of the construct reliability and replicability across studies (Hancock & Mueller, 2001), was very high for the general dimension (0.92), moderate for positive (0.78) and negative (0.71) dimensions and lower for the depressive dimension (0.41).

### Symptom dimensions in patients by a pattern of cannabis use

Models' results are presented in Table 1 which shows that:

(1) There were no differences in the distribution of positive symptoms according to early age at first use ( ⩽ 15 years old), nor, after B-H correction, according to ever or current use of cannabis. However, positive symptoms were more common among patients who spent more than 20 euros per week on cannabis (*B* = 0.3; 95% CI 0.11–0.48; *p* = 0.001).
(2) Fewer negative symptoms were observed among those patients who used cannabis at least once compared with those who never tried (*B* = −0.22; 95% CI −0.37 to −0.07; *p* = 0.004). Early age at first use and current use of cannabis was not associated with negative symptomatology.
(3) Manic symptoms were more frequent among patients who had ever used cannabis (*B* = 0.22; 95% CI 0.08–0.36; *p* = 0.002).
(4) There were no differences in the distribution of the scores on the depressive, disorganization and general psychosis dimensions according to any measure of cannabis use.

**Table 1.** Symptom dimensions in FEP patients by measures of cannabis use^a^

Symptom dimension	Ever used cannabis *B* (95% CI)	Current use of cannabis *B* (95% CI)	Age at first use of cannabis *B* (95% CI)	Money used for cannabis *B* (95% CI)
Positive	0.16* (0 to 0.31)	0.21* (0.04–0.37)	0.05 (−0.13 to 0.22)	0.3** (0.11–0.48)
Negative	−0.22** (−0.37 to −0.07)	−0.09 (−0.26 to 0.07)	0.07 (−0.09 to 0.22)	0.07 (−0.12 to 0.25)
Depressive	−0.08 (−0.24 to 0.08)	−0.08 (−0.22 to 0.06)	−0.09 (−0.23 to 0.05)	−0.11 (−0.29 to 0.06)
Disorganization	−0.01 (−0.24 to 0.03)	0.01 (−0.05 to 0.26)	0.11 (−0.06 to 0.28)	0.1 (−0.17 to 0.19)
Manic	0.22** (0.08–0.36)	0.12 (−0.02 to 0.27)	−0.09 (−0.25 to 0.07)	0.05 (−0.11 to 0.22)
General factor	0.05 (−0.06 to 0.17)	0.02 (−0.1 to 0.14)	−0.06 (−0.09 to 0.22)	0.03 (−0.11 to 0.17)

a All models were adjusted for age, sex, ethnicity, use of other recreational/illicit substances and diagnosis. Models were random-intercept models that included two random effects to allow symptomatology to vary across countries and across sites within countries but assumed that individual-level exposure to cannabis had a fixed effect across the entire sample.

Significance: *p < 0.05, **p < 0.01, ***p < 0.001; *P*-values nominally significant after Benjamini-Hochberg procedure are showed in bold.

### Psychotic experience dimensions in population controls by patterns of cannabis use

Models' results are presented in Table 2, which shows that:

(1) There were no differences in the distribution of positive psychotic experiences according to ever use of cannabis or early age at first use ( ⩽ 15 years old). However, positive psychotic experiences were more commonly reported by subjects who currently used cannabis (*B* = 0.33; 95% CI 0.15–0.51; *p* < 0.001) and who spent more than 20 euros per week on cannabis (*B* = 0.39; 95% CI 0.09–0.69; *p* = 0.011).
(2) There were no differences in the distribution of the depressive and negative experiences in population controls according to cannabis use.

**Table 2.** Psychotic experience dimensions in controls by cannabis use^a^

Psychotic experience dimension	Ever used cannabis *B* (95% CI)	Current use of cannabis *B* (95% CI)	Age at first use of cannabis *B* (95% CI)	Money used for cannabis *B* (95% CI)
Positive	0.05 (−0.06 to 0.17)	0.33*** (0.15–0.51)	0.08 (−0.11 to 0.25)	0.39* (0.09–0.69)
Negative	0.11 (−0.01 to 0.24)	0.16 (−0.03 to 0.36)	−0.11 (−0.29 to 0.07)	−0.12 (−0.2 to 0.44)
Depressive	0.09 (−0.03 to 0.21)	0.01 (−0.19 to 0.20)	−0.02 (−0.21 to 0.16)	−0.02 (−0.3 to 0.35)
General factor	0.04 (−0.08 to 0.17)	0.13 (−0.07 to 0.33)	0.08 (−0.11 to 0.22)	0.15 (−0.18 to 0.48)

a All models were adjusted for age, sex, ethnicity and use of other recreational/illicit substances. Models were random-intercept models that included two random effects to allow symptomatology to vary across countries and across sites within countries but assumed that individual-level exposure to cannabis had a fixed effect across the entire sample.

Significance: *p < 0.05, **p < 0.01, ***p < 0.001; *P*-values nominally significant after Benjamini-Hochberg procedure are showed in bold.

### Symptom dimensions by frequency of use and potency of cannabis

The independent effects of frequency of use and potency of cannabis are reported in online Supplementary Tables S6.1 and S6.2, and online Supplementary Fig. S2, showing that, only in patients, positive symptoms were more common in those who used cannabis on a daily basis and exposed to high-potency varieties

**Fig. 2.**
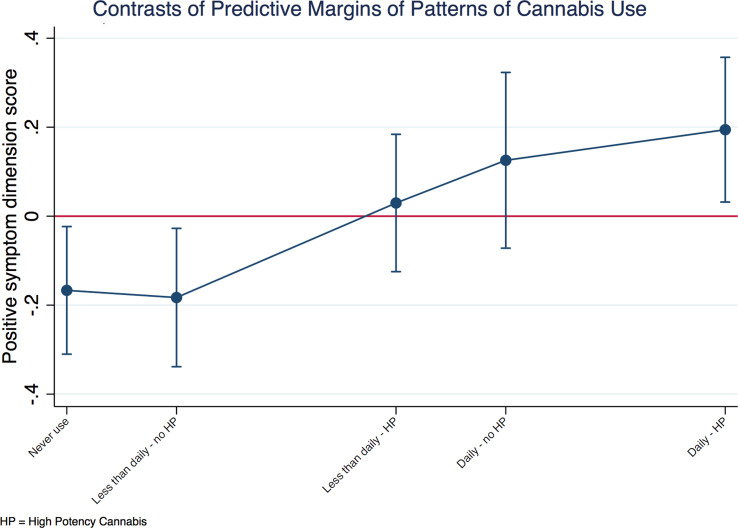
Positive symptom dimension in cases by patterns of cannabis use. Explanatory note: the positive symptom dimension predicted mean of each group of patterns of cannabis use is plotted against the predicted grand mean of all groups (represented by the red line). The positive value for the contrast of daily use of high-potency cannabis indicates more positive symptomatology in this group. On the other hand, negative values for the contrasts of the first two groups indicates less positive symptomatology when there is less exposure to cannabis. These differences are relevant, as indicated by 95% confidence intervals that do not overlap with zero. The model was a random intercept model which allowed symptoms to vary across countries and sites within countries, but it assumed that frequency of use and type of cannabis had an individual fixed effect. Values were adjusted for age, sex, ethnicity, diagnosis and use of other recreational/illicit substances.

Testing the combined ‘type-frequency’ variable in patients, we found evidence of a linear relationship between the positive symptom dimension and the extent of exposure to cannabis, with daily users of high-potency cannabis showing the highest score (*B* = 0.35; 95% CI 0.14–0.56; *p* = 0.001). Therefore, we introduced a contrast operator and plotted the exposure–response relationship for positive symptoms (Fig. 2), by comparing the predictive margins of the adjusted mean of each group against the grand adjusted mean of all groups. Figure 2 shows that the adjusted mean for daily users of high-potency cannabis was 0.2 units greater than the grand adjusted mean. Moreover, the adjusted means for the groups who never or rarely used cannabis were respectively 0.16 or 0.18 units lower than the grand adjusted mean.

**Fig. 3.**
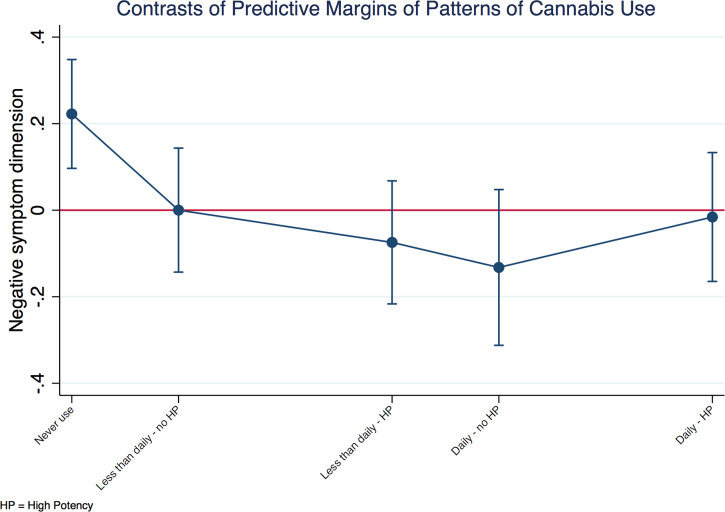
Negative symptom dimension in cases by patterns of cannabis use. Explanatory note: the negative symptom dimension predicted mean of each group of patterns of cannabis use is plotted against the predicted grand mean of all groups (represented by the red line). Subjects who had never used cannabis presented with more negative symptoms compared to the whole sample. The model was a random intercept model which allowed symptoms to vary across countries and sites within countries, but it assumed that frequency of use and type of cannabis had an individual fixed effect.

A negative relationship between the negative symptom dimension score and patterns of cannabis use was also observed in patients. Figure 3 shows that patients with psychosis who never used cannabis had more negative symptoms either compared with the grand adjusted mean or with any pattern of cannabis use.

## Discussion

### Principal findings

This is the first multinational study analysing data on the potency of cannabis and its dose-effect relationship with dimensions of symptoms in FEP patients, and dimensions of psychotic experiences in population controls. We provide the first evidence that: (1) in patients, a positive correlation exists between the extent of premorbid cannabis use and the score on the positive symptom dimension, with daily users of high-potency cannabis showing the most positive symptoms at FEP; (2) psychotic experiences in non-clinical populations are associated with the current use of cannabis but are independent of the extent of lifetime exposure to cannabis; (3) negative symptoms at FEP are more common in patients who have never tried cannabis; (4) depressive symptoms are independent of any pattern of use of cannabis.

### Limitations

Our findings must be considered in the context of two main limitations. First, individual data on patterns of cannabis use are not validated with biological samples. However, biological tests are not considered the gold standard method for such a validation (Large et al., 2012) and do not allow one to ascertain the extent of cannabis use over the years (Taylor, Sullivan, Ring, Macleod, & Hickman, 2017). Moreover, studies combining self-report and laboratory data support the reliability of subjects in reporting the type of cannabis they use (Freeman et al., 2014; Wolford et al., 1999). Second, we did not take into account the cannabidiol (CBD) contribution to the potency variable, as official data on its content in the different cannabis varieties were not available in most study sites; CBD might counterbalance Δ9-THC effects and minimize both psychotic experiences (Schubart et al., 2011) and symptoms (McGuire et al., 2018).

### Comparison with previous research

We extend previous research on cannabis and psychotic symptoms to a multinational sample confirming the association between cannabis use and positive symptoms of FEP (Ringen et al., 2016; Seddon et al., 2016). Our results are in line with Schoeler et al. (2016), who carefully scrutinized the literature on the effect of continuation of cannabis use after FEP, concluding that this would be associated with a more severe positive symptomatology (Schoeler et al., 2016). That said, any comparison with previous research is limited by the lack of information on frequency and potency in all the previous studies along with subjects' exposure to more potent varieties of cannabis in recent years (Potter et al., 2018). In this respect, we firstly provide some evidence that cannabis affects positive symptoms in a dose–response manner, further supporting the converging epidemiological and experimental evidence that the use of cannabis with high content of Δ9-THC has a more detrimental effect than other varieties (Di Forti et al., 2009; Freeman et al., 2018; Morrison et al., 2009).

We also report evidence in a multinational FEP sample of an association between lifetime cannabis use and fewer negative symptoms, the latter often considered as a marker of greater neurodevelopmental impairment in psychosis. Two opposite interpretations may be considered.

First, some authors have suggested that people suffering a psychotic disorder might abuse cannabis as an attempt to self-medicate negative symptoms, and thus the observed reduction in negative symptomatology would be an epiphenomenon due to the cannabis intake itself (Peralta & Cuesta, 1992).

Alternatively, psychotic disorders may be characterized by less neurodevelopmental features when associated with cannabis use (Ferraro et al., 2013; Ferraro et al., 2019; Murray et al., 2017; Ruiz-Veguilla, Callado, & Ferrin, 2012), hence FEP patients who do not initiate to use cannabis would present more negative symptoms.

The lack of a dose dependency in our study appears to speak against the first and in favour of the second possibility, as the difference holds between those who never obtained cannabis and those who used it only once. Moreover, negative symptoms would reduce the social and instrumental skills that were necessary to illegally obtain cannabis and sustain its use in all the countries included in the study, except Holland.

Last, we report that the cumulative exposure to cannabis does not impact on psychotic experiences in controls. One could of course argue that the largest proportion of subjects with the harmful pattern of cannabis use were patients. However, further research is needed to look into plausible mechanisms of resilience to the psychotogenic effect of cannabis as observed in our controls, who report psychotic experiences if current users but do not seem to accumulate a risk over life time cannabis use and develop psychotic disorders. Indeed, future studies should aim to: (1) investigate if and how genetic factors, plausibly regulating the endocannabinoid and dopamine systems, pose a small subset of cannabis users at high risk of developing a psychotic disorders with particular symptomatology; (2) clarify over the course of the disorder whether differences in symptomatology between current and former cannabis users may be related to residual cannabis effects.

### Implications

The novelty of our study is based on our examination of data on lifetime frequency of cannabis use and on the type of the cannabis used; the availability of high-potency types is increasing worldwide. For instance, a recent potency study revealed that in London, the high-potency type of cannabis called skunk has now taken up 96% of the street market (Potter et al., 2018). The EMCDDA has described a European cannabis market characterized by potent varieties (European Monitoring Centre for Drugs and Drug Addiction, 2013) like those present in Amsterdam coffee shops that can reach up to 39% of THC. Indeed, as daily use of high-potency cannabis has been associated with high rates of psychotic disorders across Europe (Di Forti et al., 2019), here we show that in FEP patients daily use of high-potency cannabis drives a high score on the positive symptom dimension. Further research should aim to determine biological mechanisms underlying how cannabis contribute to a particular clinical presentation of psychosis. Meanwhile, translating current findings into clinical practice, symptom dimension scores can be used to stratify patients and develop secondary prevention schemes for cannabis-associated psychosis.

## Supplementary material

For supplementary material accompanying this paper visit https://doi.org/10.1017/S0033291720000082. click here to view supplementary material

## EU-GEI group authorship includes

Kathryn Hubbard^1^, Stephanie Beards^1^, Simona A. Stilo^2^, Mara Parellada^3^, David Fraguas^3^, Marta Rapado Castro^3^, Álvaro Andreu-Bernabeu^3^, Gonzalo López^3^, Mario Matteis^3^, Emiliano González^3^, Manuel Durán-Cutilla^3^, Covadonga M. Díaz-Caneja^3^, Pedro Cuadrado^4^, José Juan Rodríguez Solano^5^, Angel Carracedo^6^, Javier Costas^6^, Emilio Sánchez^7^, Silvia Amoretti^8^, Esther Lorente-Rovira^9^, Paz Garcia-Portilla^10^, Estela Jiménez-López^11^, Nathalie Franke^12^, Daniella van Dam^12^, Fabian Termorshuizen^13,14^, Nathalie Franke^13^, Elsje van der Ven^13,14^, Elles Messchaart^14^, Marion Leboyer^15,16,17,18^, Franck Schürhoff^15,16,17,18^, Stéphane Jamain^16,17,18^, Grégoire Baudin^15,16^, Aziz Ferchiou^15,16^, Baptiste Pignon^15,16,18^, Jean-Romain Richard^16,18^, Thomas Charpeaud^18,19,21^, Anne-Marie Tronche^18,19,21^, Flora Frijda^20^, Giovanna Marrazzo^23^, Lucia Sideli^22^, Crocettarachele Sartorio^22,23^, Fabio Seminerio^22^, Camila Marcelino Loureiro^24,25^, Rosana Shuhama^24,25^, Mirella Ruggeri^26^, Chiara Bonetto^26^, Doriana Cristofalo^26^, Marco Seri^27^, Giuseppe D'Andrea^27^, Michael C O'Donovan^28^, Alexander L Richards^28^

## Notes

^1^Department of Health Service and Population Research, Institute of Psychiatry, King's College London, De Crespigny Park, Denmark Hill, London, United Kingdom, SE5 8AF

^2^Department of Psychosis Studies, Institute of Psychiatry, King's College London, De Crespigny Park, Denmark Hill, London, United Kingdom SE5 8AF

^3^Department of Child and Adolescent Psychiatry, Hospital General Universitario Gregorio Marañón, School of Medicine, Universidad Complutense, IiSGM (CIBERSAM), C/Doctor Esquerdo 46, 28007 Madrid, Spain

^4^Villa de Vallecas Mental Health Department, Villa de Vallecas Mental Health Centre, Hospital Universitario Infanta Leonor / Hospital Virgen de la Torre, C/San Claudio 154, 28038 Madrid, Spain

^5^Puente de Vallecas Mental Health Department, Hospital Universitario Infanta Leonor / Hospital Virgen de la Torre, Centro de Salud Mental Puente de Vallecas, C/Peña Gorbea 4, 28018 Madrid, Spain

^6^Fundación Pública Galega de Medicina Xenómica, Hospital Clínico Universitario, Choupana s/n, 15782 Santiago de Compostela, Spain

^7^Department of Psychiatry, Hospital General Universitario Gregorio Marañón, School of Medicine, Universidad Complutense, IiSGM (CIBERSAM), C/Doctor Esquerdo 46, 28007 Madrid, Spain

^8^Department of Psychiatry, Hospital Clinic, IDIBAPS, Centro de Investigación Biomédica en Red de Salud Mental (CIBERSAM), Universidad de Barcelona, C/Villarroel 170, escalera 9, planta 6, 08036 Barcelona, Spain

^9^Department of Psychiatry, School of Medicine, Universidad de Valencia, Centro de Investigación Biomédica en Red de Salud Mental (CIBERSAM), C/Avda. Blasco Ibáñez 15, 46010 Valencia, Spain

^10^Department of Medicine, Psychiatry Area, School of Medicine, Universidad de Oviedo, Centro de Investigación Biomédica en Red de Salud Mental (CIBERSAM), C/Julián Clavería s/n, 33006 Oviedo, Spain

^11^Department of Psychiatry, Servicio de Psiquiatría Hospital ‘Virgen de la Luz’, C/Hermandad de Donantes de Sangre, 16002 Cuenca, Spain

^12^Department of Psychiatry, Early Psychosis Section, Academic Medical Centre, University of Amsterdam, Meibergdreef 5, 1105 AZ Amsterdam, The Netherlands

^13^Rivierduinen Centre for Mental Health, Leiden, Sandifortdreef 19, 2333 ZZ Leiden, The Netherlands

^14^Department of Psychiatry and Neuropsychology, School for Mental Health and Neuroscience, South Limburg Mental Health Research and Teaching Network, Maastricht University Medical Centre, P.O. Box 616, 6200 MD Maastricht, The Netherlands

^15^AP-HP, Groupe Hospitalier ‘Mondor’, Pôle de Psychiatrie, 51 Avenue de Maréchal de Lattre de Tassigny, 94010 Créteil, France

^16^INSERM, U955, Equipe 15, 51 Avenue de Maréchal de Lattre de Tassigny, 94010 Créteil, France

^17^Faculté de Médecine, Université Paris-Est, 51 Avenue de Maréchal de Lattre de Tassigny, 94010 Créteil, France

^18^Fondation Fondamental, 40 Rue de Mesly, 94000 Créteil, France

^19^CMP B CHU, BP 69, 63003 Clermont Ferrand, Cedex 1, France

^20^Etablissement Public de Santé Maison Blanche, Paris, France

^21^Université Clermont Auvergne, EA 7280, Clermont-Ferrand, 63000, France

^22^Department of Experimental Biomedicine and Clinical Neuroscience, Section of Psychiatry, University of Palermo, Via G. La Loggia n.1, 90129 Palermo, Italy

^23^Unit of Psychiatry, ‘P. Giaccone’ General Hospital, Via G. La Loggia n.1, 90129 Palermo, Italy

^24^Departamento de Neurociências e Ciencias do Comportamento, Faculdade de Medicina de Ribeirão Preto, Universidade de São Paulo, Av. Bandeirantes, 3900 -Monte Alegre- CEP 14049-900, Ribeirão Preto, SP, Brasil

^25^Núcleo de Pesquina em Saúde Mental Populacional, Universidade de São Paulo, Avenida Doutor Arnaldo 455, CEP 01246-903, SP, Brasil

^26^Section of Psychiatry, Department of Neuroscience, Biomedicine and Movement, University of Verona, Piazzale L.A. Scuro 10, 37134 Verona, Italy

^27^Department of Medical and Surgical Science, Psychiatry Unit, Alma Mater Studiorum Università di Bologna, Viale Pepoli 5, 40126 Bologna, Italy

^28^Division of Psychological Medicine and Clinical Neurosciences, MRC Centre for Neuropsychiatric Genetics and Genomics, Cardiff University, Cardiff CF24 4HQ, UK
