# Supplementary material for: Daily use of high-potency cannabis is associated with more positive symptoms in first-episode psychosis patients: the EU-GEI case–control study
Source: Psychol Med. 2020 Mar 18;51(8):1329–37. doi: 10.1017/S0033291720000082 (PMC8223239; doi:10.1017/S0033291720000082)
Supplement: Supplementary file 1 [file S0033291720000082sup001.docx]

**SUPPLEMENTARY MATERIAL**

**Supplementary Table S1. Socio-demographic characteristics and history of substance misuse of the analysed sample**

|  | **FEP**  **N=901** | **Controls**  **N=1235** |
| --- | --- | --- |
| **Age** (mean; SD) | 30.8 (10.5) | 36.1 (13.3) |
| **Sex** (male %; N) | 61.9 (558) | 47 (581) |
|  | | |
| Self-reported Ethnicity |  |  |
| **White** (%; N) | 59.05 (532) | 75.22 (929) |
| **Black** | 18.65 (168) | 9.55 (118) |
| **Mixed** | 11.54 (104) | 9.15 (113) |
| **Asian** | 3.55 (32) | 2.67 (33) |
| **North African** | 4.66 (42) | 1.86 (23) |
| **Others** | 2.55 (23) | 1.54 (19) |
|  | | |
| Ever used cannabis |  |  |
| **Yes** (%; N) | 64.93 (585) | 46.48 (574) |
| Missing | 1.44 (13) | 1.05 (13) |
|  |  |  |
| Current use of cannabis |  |  |
| **Yes** (%; N) | 21.64 (195) | 10.61 (131) |
| Missing | 1.78 (16) | 1.05 (13) |
|  |  |  |
| Age at first use of cannabis |  |  |
| **Never Used** (%; N) | 33.63 (303) | 52.47 (648) |
| **<=15 year old** | 27.75 (250) | 13.52 (167) |
| **16 year old and older** | 35.74 (322) | 32.96 (407) |
| Missing | 2.89 (26) | 1.05 (13) |
|  |  |  |
| Money used for cannabis (weekly) |  |  |
| **From 0 to 20 euro** | 76.47 (689) | 92.3 (1,140) |
| **More than 20 euro** | 16.1 (145) | 3.16 (39) |
| Missing | 7.44 (67) | 4.53 (56) |
|  |  |  |
| Lifetime frequency of use |  |  |
| **Never use** | 56.83 (512) | 52.47 (648) |
| **Less than daily** | 11.54 (104) | 39.68 (490) |
| **Daily** | 28.86 (260) | 6.72 (83) |
| Missing | 2.77 (25) | 1.13 (14) |
|  |  |  |
| Type of cannabis |  |  |
| **Never used** | 33.63 (303) | 55.57 (648) |
| **Less than 10% THC** | 26.64 (240) | 23.89 (295) |
| **More than 10% THC** | 32.63 (294) | 18.06 (223) |
| Missing | 7.1 (64) | 5.59 (69) |
|  |  |  |
| Current tobacco use |  |  |
| **>10 cigarettes x day** (%; N) | 28.71 (262) | 10.85 (134) |
| Missing | 3.77 (34) | 1.94 (24) |
|  |  |  |
| Current use of other drugs |  | |
| **Stimulants** (%; N)  Missing | 8.62 (82)  1.6 (15) | 4.53 (56)  1.05 (13) |
| **Hallucinogens**  Missing | 5.23 (49)  1.92 (18) | 2.02 (25)  1.21 (15) |
| **Ketamine**  Missing | 2.13 (20)  1.92 (18) | 1.05 (13)  1.21(15) |
| **Novel Psychoactive Substances**  Missing | 1.39 (13)  1.71 (16) | 0.65 (8)  1.05 (13) |
| **Crack**  Missing | 2.67 (25)  1.6 (15) | 2 (0.16)  1.05 (13) |
| **Cocaine**  Missing | 14.94 (140)  1.81 (17) | 5.83 (72)  1.13 (14) |
|  |  |  |
| Current alcohol overuse |  |  |
| **Drinks =>10 units per week** (%; N)  Missing | 10.88 (98)  11.4(103) | 12.47 (154)  3.24(40) |
|  |  |  |
| Diagnosis |  |  |
| **Schizophrenia** (%;N) | 13.2 (282) |  |
| **Schizoaffective disorders** | 17.84 (381) |  |
| **Bipolar Disorders** | 2.48 (53) |  |
| **Psychotic Depression** | 1.92 (41) |  |
| **Unspecified Psychosis** | 6.74 (144) |  |
|  |  |  |

**Supplementary table S2. Cannabis measures in the EU-GEI study**

| **Lifetime cannabis use** | 0=*never used* | 1=*Yes* |  |
| --- | --- | --- | --- |
| **Currently using cannabis** | 0=*no use at the time of recruitment in the study and over the previous 4 weeks* | 1=Yes |  |
| **Age at first use** **of cannabis** | 0=*started at age 16 years or older* | *1=started at age 15 years or younger* |  |
| **Lifetime frequency of use** | *0=never used* | *1=used less than daily* | *2=used daily* |
| **Money spent weekly on cannabis** | *0=never used or spent 20 EURO or less per week* | *1= spent more*  *than 20 EURO per week* |  |
| **Type of cannabis used^1^** | *0= never used* | *1= types with THC<10%* | *2= types with THC=>10%* |

*^1^Explanatory note: The potency variable was defined by a cut off of 10% of the THC concentration expected in the different varieties of cannabis in each catchment area, based on government and national data examined by the European Monitoring Centre for Drugs and Drug Addiction (EMCDDA) (European Monitoring Centre for Drugs and Drug Addiction, 2013, Di Forti et al., 2019).*

*Cannabis varieties classified as low potency (THC<10%) were: hash/resin from UK and Italy, imported herbal cannabis from UK, Italy, Spain and France, Brazilian marijuana and hash and the Dutch Geimporteerde Wiet.*

*Cannabis varieties classified as high-potency (THC>10%) were: UK home-grown skunk/sensimilla UK Super Skunk, Italian home-grown skunk/sensimilla, Italian Super Skunk, the Dutch Nederwiet, Nederhasj and geimporteerde hasj, the Spanish and French Hashish (from Morocco), Spanish home-grown sensimilla, French home-grown skunk/sensimilla/super-skunk and Brazilian skunk.*

**Supplementary table S3. Prevalence of CAPE psychotic experiences in population controls**

**Supplementary Figure S1**

**Path diagrams of the five psychotic experiences’ models**

Model A


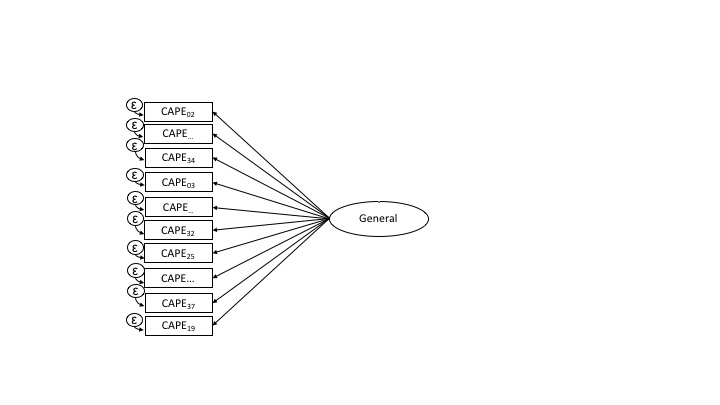


Model B

*
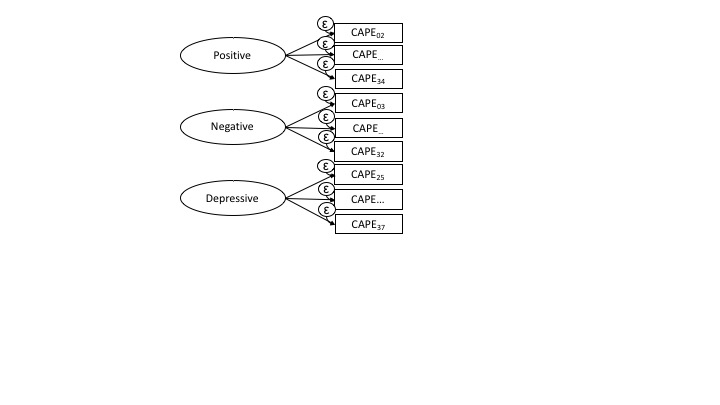
*

Model C

*
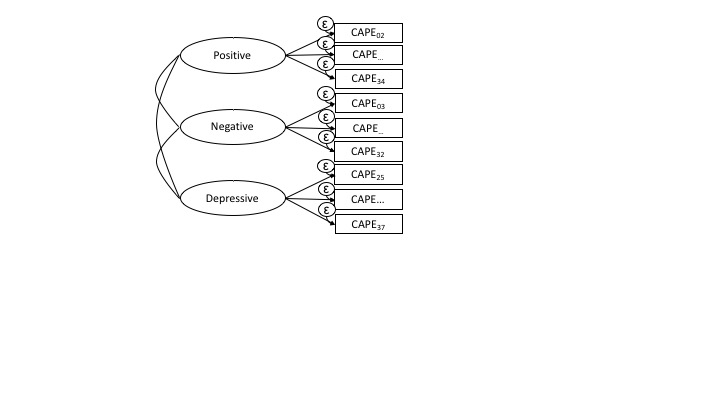
*

Model D

*
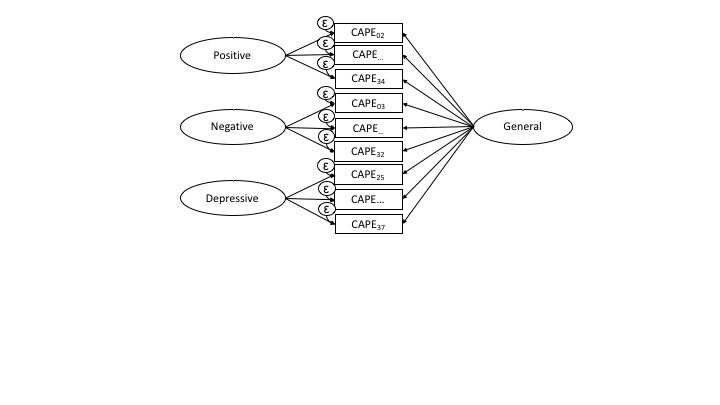
*

Model E

*
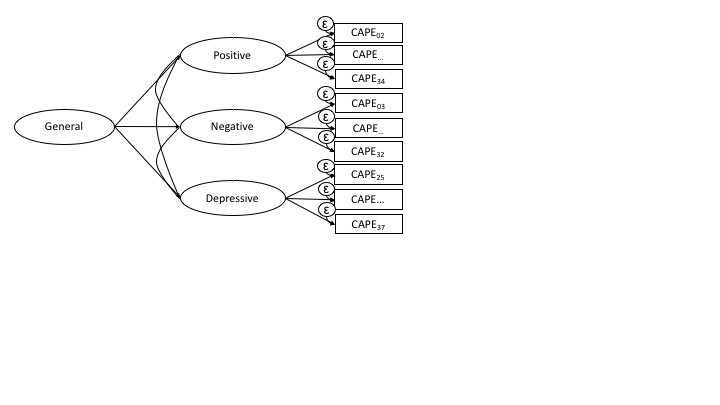
*

Explanatory note: (▭) Observed symptoms (CAPE items); (Ö) Unobserved variables (latent factors); (⭢) item loading on latent factors; (ε) item error variance. CAPE item numbers are showed in Tables S1; for simplicity, only three items for each latent factor are presented in the diagrams.

Explanatory note: *Model A*: unidimensional model with one unique general factor; *Model B*: multidimensional model with three uncorrelated specific factors; *Model C*: multidimensional model with three correlated specific factors; *Model D*: bifactor model with one general factor and three uncorrelated specific factors; Model *E*: hierarchical model with three correlated first-order specific factors and one general second-order factor.

As showed in the main text and in Table 1, the bifactor model for the CAPE (Model D) best reflected the dimensional structure of psychosis in population controls when compared with the other models. This is consistent with our previous findings on the bifactor model for the OPCRIT in patients (Quattrone *et al.*, 2019). The bifactor model allows examining the variance due to each dimension whilst partitioning out the variance due to the common item effect of the whole symptomatology. Thus, in this study, we performed the best possible evaluation of the impact of cannabis use on specific subsets of psychotic symptoms or experiences in patients and controls.

**Supplementary Table S4. Model fit statistics of unidimensional, multidimensional, bifactor, second-order models for psychotic experiences and for psychotic symptoms**

| CAPE (CONTROLS) | | | | |
| --- | --- | --- | --- | --- |
|  | Full information fit statistics^a^ | | | |
|  | LL | AIC | BIC | SABIC |
| A - Unidimensional Model | -23638 | 47397 | 47715 | 47524 |
| B - Multidimensional Model (three uncorrelated factors) | -23844 | 47808 | 48126 | 47936 |
| C - Multidimensional Model (three correlated factors) | -23341 | 46808 | 47142 | 46942 |
| **D - Bifactor Model (one general factor and three specific uncorrelated factors)** | **-23139** | **46458** | **46935** | **46649** |
| E - Hierarchical Model (three first-order specific correlated factors and one second order general factor) | -23341 | 46807 | 47135 | 46938 |
|  | | | | |
| OPCRIT (PATIENTS) (Quattrone *et al.*, 2019) | | | | |
|  | Full information fit statistics^a^ | | | |
|  | LL | AIC | BIC | SABIC |
| A - Unidimensional Model | -29965 | 60126 | 60618 | 60306 |
| B - Multidimensional Model (five uncorrelated factors) | -28070 | 56335 | 56826 | 56515 |
| C - Multidimensional Model (five correlated factors) | -27894 | 56004 | 56546 | 56202 |
| **D - Bifactor Model (one general factor and five specific uncorrelated factors)** | **-27597** | **55489** | **56226** | **55759** |
| E - Hierarchical Model (five first-order specific correlated factors and one second order general factor) | -27995 | 56197 | 56713 | 56386 |

LL, log-likelihood; AIC, Akaike Information Criterion; BIC, Bayesian Information Criterion; SABIC Sample-size Adjusted Bayesian Information Criterion

A difference of 10 in AIC, BIC and SABIC is considered important. Lower values indicate a statistically better model fit (best values across models are indicated in bold).

**Supplementary Table S5. Prevalence of OPCRIT symptoms in patients (Quattrone *et al.*, 2019)**

| **OPCRIT ITEM** | **Item no.** | **Factor** | **Valid frequency** |
| --- | --- | --- | --- |
| Persecutory Delusions | 54 | POS | 71.6% (794) |
| Well organised delusions | 55 | POS | 41.6% (458) |
| Delusions of influence | 58 | POS | 24.1% (267) |
| Bizarre Delusions | 59 | POS | 23.3% (259) |
| Widespread Delusions | 60 | POS | 42.4% (437) |
| Delusions of passivity | 61 | POS | 15.2% (168) |
| Primary delusional perception | 62 | POS | 26.2% (286) |
| Other primary delusions | 63 | POS | 19.4% (213) |
| Delusions & hallucinations last for one week | 64 | POS | 47.9% (495) |
| Persecutory delusions & hallucinations | 65 | POS | 30.1% (311) |
| Thought insertion | 66 | POS | 16.4% (180) |
| Thought broadcast | 68 | POS | 15.5% (171) |
| Third person auditory hallucinations | 73 | POS | 29.3% (322) |
| Running commentary voices | 74 | POS | 24.1% (266) |
| Abusive/accusatory/persecutory voices | 75 | POS | 31.8% (329) |
| Other (non-affective) auditory hallucinations | 76 | POS | 23.3% (264) |
| Non-affective hallucination in any modality | 77 | POS | 26.7% (294) |
| Negative formal thought disorder | 29 | NEG | 19% (209) |
| Restricted affect | 32 | NEG | 36.4% (404) |
| Blunted affect | 33 | NEG | 21.9% (243) |
| Bizarre behaviour | 17 | DIS | 44.9% (496) |
| Speech difficult to understand | 26 | DIS | 20.9% (230) |
| Incoherent | 27 | DIS | 13% (13) |
| Positive formal thought disorder | 28 | DIS | 24.3% (268) |
| Inappropriate affect | 34 | DIS | 19.6% (216) |
| Excessive activity | 19 | MAN | 25.5% (283) |
| Reckless activity | 20 | MAN | 21% (233) |
| Distractibility | 21 | MAN | 47.4% (521) |
| Reduced need for sleep | 22 | MAN | 30.8% (340) |
| Agitated activity | 23 | MAN | 41.3% (457) |
| Pressured speech | 30 | MAN | 23% (255) |
| Thoughts racing | 31 | MAN | 33% (365) |
| Elevated mood | 35 | MAN | 20.6% (229) |
| Irritable mood | 36 | MAN | 47.7% (529) |
| Increased self esteem | 56 | MAN | 24.1% (267) |
| Grandiose Delusions | 57 | MAN | 23.3% (259) |
| Slowed activity | 24 | DEP | 23.6% (261) |
| Loss of energy/tiredness | 25 | DEP | 40.1% (444) |
| Dysphoria | 37 | DEP | 48.7% (540) |
| Loss of pleasure | 39 | DEP | 43.2% (477) |
| Poor concentration | 41 | DEP | 61% (676) |
| Excessive self-reproach | 42 | DEP | 25.8% (286) |
| Suicidal ideation | 43 | DEP | 34.2% (380) |
| Initial insomnia | 44 | DEP | 52.4% (576) |
| Middle insomnia (broken sleep) | 45 | DEP | 38.4% (423) |
| Early morning waking | 46 | DEP | 24.9% (274) |
| Excessive sleep | 47 | DEP | 15.2% (168) |
| Poor appetite | 48 | DEP | 37% (407) |
| Weight Loss | 49 | DEP | 29.3% (315) |

**Supplementary Table S6.1. Symptom dimensions in patients by frequency of use and potency of cannabis^a^**

| **Model** | **Lifetime frequency of use**  **B (95% CI)** | | **Potency of cannabis**  **B (95% CI)** | |
| --- | --- | --- | --- | --- |
|  | Less than daily  *(v. never used)* | Daily  *(v. never used)* | low potency  *(v. no use)* | high potency  *(v. no use)* |
| Positive symptom dimension | 0.1  (-0.21 to 0.22) | **0.23****  (0.07 to 0.39) | 0.09  (-0.12 to 0.28) | **0.22****  (0.02 to 0.29) |
| Negative symptom dimension | -0.07  (-0.29 to 0.15) | -0.09  (-0.26 to 0.09) | **-0.24****  (-0.41 to -0.06) | -0.2*  (-0.39 to -0.02) |
| Depressive symptom dimension | -0.12  (-0.31 to 0.06) | -0.1  (-0.24 to 0.04) | -0.13  (-0.28 to 0.03) | -0.13  (-0.29 to 0.03) |
| Disorganization symptom dimension | 0.26*  (0.05 to 0.47) | 0.11  (-0.04 to 0.27) | -0.02  (-0.19 to 0.15) | 0.13  (-0.04 to 0.32) |
| Manic symptom dimension | 0.02  (-0.17 to 0.22) | 0.13  (-0.02 to 0.28) | **0.23****  (0.06 to 0.39) | **0.27****  (0.1 to 0.44) |
| General  Psychosis factor | 0.17*  (0.01 to 0.33) | 0.12*  (0.01 to 0.25) | 0.06  (-0.07 to 0.19) | 0.02  (-0.12 to 0.17) |

^a^All models were adjusted for age, sex, ethnicity, current use of other recreational/illicit substances, and diagnosis. Models were random-intercept models that included two random effects to allow symptomatology to vary across countries and across sites within countries but assumed that individual-level exposure to cannabis had a fixed effect across the entire sample.

Significance: * p < 0.05, ** p < 0.01, *** p < 0.001; associations that hold after Benjamini-Hochberg procedure are showed in bold.

**Supplementary Table S6.2. Psychotic experience dimensions in controls by frequency of use and potency of cannabis^a^**

| **Model** | **Lifetime frequency of use**  **B (95% CI)** | | **Potency of cannabis**  **B (95% CI)** | |
| --- | --- | --- | --- | --- |
|  | Less than daily  *(v. never used)* | Daily use  *(v. rare and never use)* | Low Potency  *v. no use* | High potency  *v. no use* |
| Positive psychotic experience dimension | 0.04  (-0.08 to 0.16) | 0.17  (-0.05 to 0.38) | 0.08  (-0.06 to 0.22) | 0.03  (-0.13 to 0.19) |
| Negative experience dimension | 0.11  (-0.02 to 0.24) | 0.14  (-0.09 to 0.38) | 0.09  (-0.05 to 0.24) | 0.12  (-0.05 to 0.29) |
| Depressive  experience dimension | 0.08  (-0.05 to 0.2) | 0.17  (-0.08 to 0.4) | 0.08  (-0.07 to 0.23) | 0.05  (-0.11 to 0.22) |
| General psychotic experience factor | 0.03  (-0.1 to 0.16) | 0.13  (-0.11 to 0.37) | 0.08  (-0.07 to 0.23) | -0.02  (-0.19 to 0.15) |

^a^All models were adjusted for age, sex, ethnicity, current use of other recreational/illicit substances. Models were random-intercept models that included two random effects to allow symptomatology to vary across countries and across sites within countries but assumed that individual-level exposure to cannabis had a fixed effect across the entire sample.

Significance: * p < 0.05, ** p < 0.01, *** p < 0.001

**Supplementary Figure S2. Symptom dimensions by frequency of use and potency of cannabis**

**
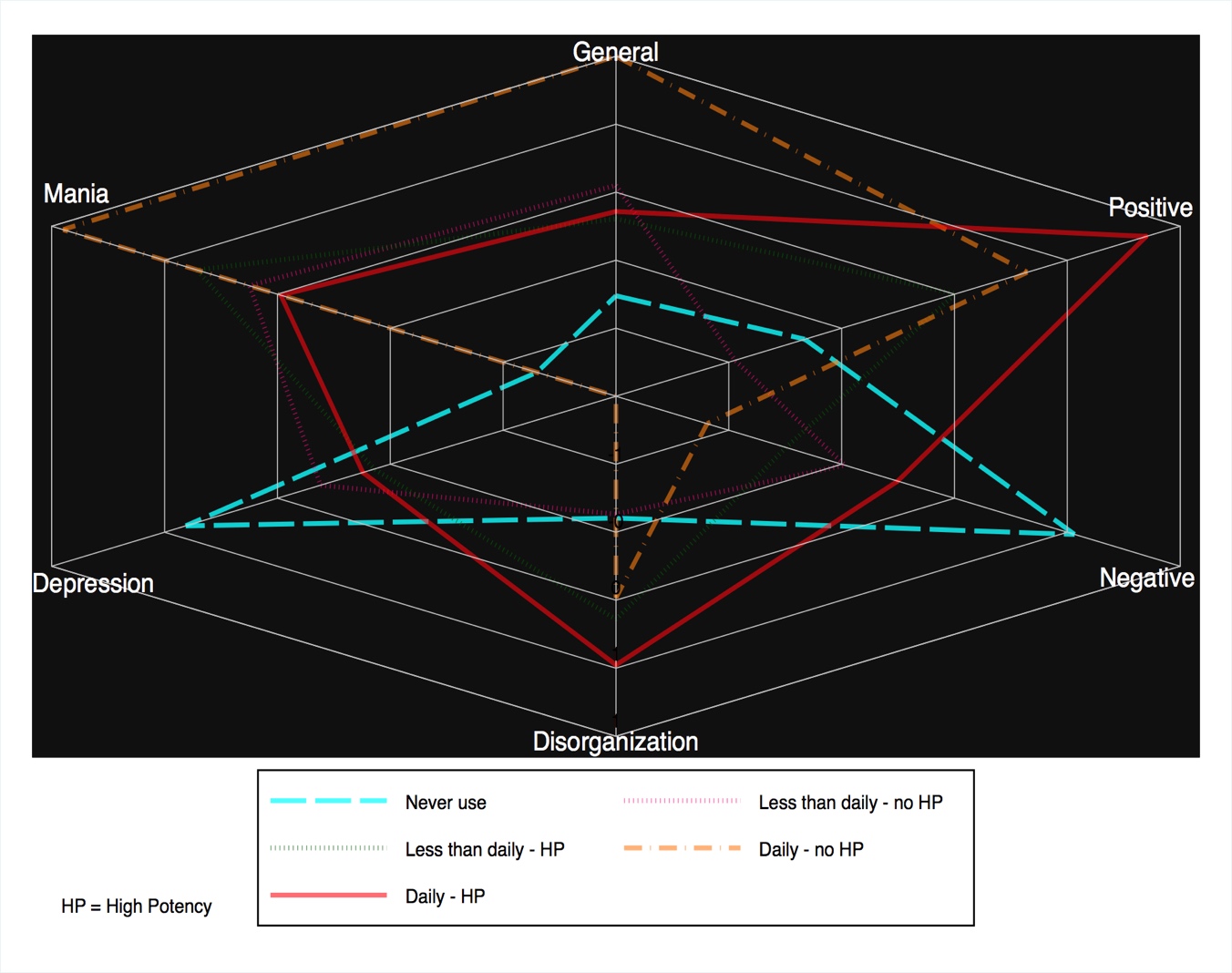
**

**References**

**Di Forti, M., Quattrone, D., Freeman, T. P., Tripoli, G., Gayer-Anderson, C., Quigley, H., . . . van der Ven, E.** (2019). The contribution of cannabis use to variation in the incidence of psychotic disorder across Europe (EU-GEI): a multicentre case-control study. *The Lancet Psychiatry* **6**, 427-436.

**European Monitoring Centre for Drugs and Drug Addiction** (2013). *European drug report: trends and developments*. Luxembourg: Publications Office of the European Union, 2013.

**Quattrone, D., Di Forti, M., Gayer-Anderson, C., Ferraro, L., Jongsma, H. E., Tripoli, G., . . . Reininghaus, U.** (2019). Transdiagnostic dimensions of psychopathology at first episode psychosis: findings from the multinational EU-GEI study. *Psychol Med* **49**, 1378-1391.
